# Online Alcohol Interventions: A Systematic Review

**DOI:** 10.2196/jmir.1479

**Published:** 2010-12-19

**Authors:** Angela White, David Kavanagh, Helen Stallman, Britt Klein, Frances Kay-Lambkin, Judy Proudfoot, Judy Drennan, Jason Connor, Amanda Baker, Emily Hines, Ross Young

**Affiliations:** ^7^School of Advertising, Marketing and Public RelationsFaculty of BusinessQueensland University of TechnologyQueenslandAustralia; ^6^BlackDog InstituteSchool of PsychiatryUniversity of New South WalesSydneyAustralia; ^5^Centre for Brain and Mental Health ResearchFaculty of HealthUniversity of NewcastleNewcastleAustralia; ^4^National Drug and Alcohol Research CentreUniversity of New South WalesSydneyAustralia; ^3^National eTherapy CentreFaculty of Life and Social SciencesSwinburne UniversityMelbourneAustralia; ^2^Institute of Health & Biomedical Innovation and School of Psychology & CounsellingQueensland University of TechnologyBrisbaneAustralia; ^1^Centre for Youth Substance Abuse ResearchFaculty of Health SciencesThe University of QueenslandQueenslandAustralia

**Keywords:** Alcohol, drugs, Internet, physical health, website interactivity, online treatment, online information

## Abstract

**Background:**

There has been a significant increase in the availability of online programs for alcohol problems. A systematic review of the research evidence underpinning these programs is timely.

**Objectives:**

Our objective was to review the efficacy of online interventions for alcohol misuse. Systematic searches of Medline, PsycINFO, Web of Science, and Scopus were conducted for English abstracts (excluding dissertations) published from 1998 onward. Search terms were: (1) Internet, Web*; (2) online, computer*; (3) alcohol*; and (4) E\effect*, trial*, random* (where * denotes a wildcard). Forward and backward searches from identified papers were also conducted. Articles were included if (1) the primary intervention was delivered and accessed via the Internet, (2) the intervention focused on moderating or stopping alcohol consumption, and (3) the study was a randomized controlled trial of an alcohol-related screen, assessment, or intervention.

**Results:**

The literature search initially yielded 31 randomized controlled trials (RCTs), 17 of which met inclusion criteria. Of these 17 studies, 12 (70.6%) were conducted with university students, and 11 (64.7%) specifically focused on at-risk, heavy, or binge drinkers. Sample sizes ranged from 40 to 3216 (median 261), with 12 (70.6%) studies predominantly involving brief personalized feedback interventions. Using published data, effect sizes could be extracted from 8 of the 17 studies. In relation to alcohol units per week or month and based on 5 RCTs where a measure of alcohol units per week or month could be extracted, differential effect sizes to posttreatment ranged from 0.02 to 0.81 (mean 0.42, median 0.54). Pre-post effect sizes for brief personalized feedback interventions ranged from 0.02 to 0.81, and in 2 multi-session modularized interventions, a pre-post effect size of 0.56 was obtained in both. Pre-post differential effect sizes for peak blood alcohol concentrations (BAC) ranged from 0.22 to 0.88, with a mean effect size of 0.66.

**Conclusions:**

The available evidence suggests that users can benefit from online alcohol interventions and that this approach could be particularly useful for groups less likely to access traditional alcohol-related services, such as women, young people, and at-risk users. However, caution should be exercised given the limited number of studies allowing extraction of effect sizes, the heterogeneity of outcome measures and follow-up periods, and the large proportion of student-based studies. More extensive RCTs in community samples are required to better understand the efficacy of specific online alcohol approaches, program dosage, the additive effect of telephone or face-to-face interventions, and effective strategies for their dissemination and marketing.

## Introduction

The World Health Organization (WHO) has estimated that there are about 2 billion people worldwide who consume alcoholic beverages and 76.3 million with diagnosable alcohol use disorders [1]. Alcohol use is related to a wide range of physical, mental, and social harms [1], with harmful use ranked as the fifth leading risk factor for premature death and disability in the world [2]. Alcohol is estimated to be responsible for 3.8% of deaths and 4.6% of disability-adjusted life years lost worldwide, costing more than 1% of the gross national product of middle-income countries [3]. These disorders can negatively impact on social functioning [4] and contribute to fatalities and injuries related to drinking and driving, reduced job performance and absenteeism, aggressive behavior, and family and other relationship conflicts [5]. Tragically, young people are significantly affected, with 18 to 35 year olds having the highest peak consumption and the greatest risk of short-term harm [6,7].

The size of the community-wide challenges posed by alcohol consumption has triggered a substantial body of research into brief, low-cost interventions. These interventions have demonstrated efficacy [8] and informed the way in which services are delivered at primary care and specialist levels. However, there remains a need to engage people with risky levels of drinking or low-level problems who are unwilling to or simply do not seek assistance through traditional health services or self-help groups [8]. Compounding this issue is a lack of health care professionals who routinely deliver effective interventions for alcohol misuse, especially in rural and remote areas [9,10]. Previous research on alcohol interventions by mail or other bibliotherapy approaches have shown these delivery methods to be effective [11,12], but there are delays in providing timely support and feedback by mail unless these are used in conjunction with telephone support.

In 2009 it was estimated that over a quarter of the world’s population used the Internet [13], with 18 to 32 year olds representing 30% of all adult Internet users [14]. Given that the peak age for binge drinking is 20 to 29 years [7] and that young people are underrepresented in users of standard face-to-face alcohol and other drug (AOD) specialist services, the Internet could be an effective medium to engage this population. In fact, 14% of young adult Internet users in the United States (18 to 29 year olds) have searched the Internet for information concerning alcohol or drug problems [15].

Several interactive computer-based alcohol screening and intervention programs have been developed to be delivered either through stand-alone computers [16] or via the Internet. Current Internet programs range from user-generated content applications such as Web logs/blogs, Web-based instant messaging technologies, or discussion boards (eg, AlcoholHelpCenter.net [17]), to interactive software applications. Even within interactive applications there is substantial variability, from brief normative feedback interventions [18] to multi-session modularized programs (eg, AlcoholEdu [19]) and psychotherapy substance mediation services involving a therapist [20,21]. Many of these program applications include brief intervention strategies and educational content based on a harm-reduction philosophy [22] and motivational interviewing techniques that are presented in a self-help workbook style [23].

Much of the published literature concerning online alcohol interventions has been descriptive [24], providing general information on program evolution, application, acceptability, and usage [17,25]. Several studies of problematic drinkers confirm the acceptability of online alcohol screening and intervention [26,27], and usage data confirm that these types of websites are accessed by numbers of users that would overwhelm traditional face-to-face services. Linke et al [28] reported that the alcohol-specific intervention website for heavy drinkers, Down Your Drink, had an average of 1039 visits per month (range 706 to 1541) or 34 visits per day (range 25 to 49), with 1319 people from 41 countries registering with the online program over a 6-month period.

Internet available AOD information and services have considerable reach and are often accessed by populations who do not necessarily access standard AOD services. For example, over half of the users of the 6-week Down Your Drink Internet intervention were women [29] as were 61% of individuals who accessed the online self-assessment tool, Drinking Habit Test [30]. One of the most commonly cited reasons for using online AOD health resources has been their 24-hour accessibility [29] unconstrained by geographic locale. Other reasons include ease of access to a computer, the anonymity and privacy afforded by the medium, and not having to attend face-to-face meetings [31,32].

The Internet is a medium that is increasingly being used to deliver alcohol resources and services. In parallel with this has been burgeoning research on the Internet’s impact, with an increasing number of studies now being published in this area. It is, therefore, timely to assess the current status of the efficacy of online alcohol intervention programs to inform both the clinical application of such interventions, as well as identify directions for future research.

## Method

### Literature Search and Selection of Studies

Relevant articles published in English from 1998 up to and including December 2009 were identified through electronic searches of Medline, PsycINFO, Web of Science and Scopus databases. The following terms were used in the search: (1) Internet, Web*; (2) online, computer*; (3) alcohol*; and (4) E\effect*, trial*, random* (where * denotes the relevant wildcard for the database). Titles and abstracts of all potentially relevant articles were independently reviewed for possible inclusion by 3 of the authors (AW, HS, DK). Articles were included if (1) the primary intervention was delivered and accessed via the Internet (including password-protected sites), (2) the intervention focused on moderating or stopping alcohol consumption, and (3) the study was a randomized controlled trial of an alcohol-related screen, assessment, or intervention. Unpublished dissertations were not included.

### Data Extraction and Analysis

Data extraction was carried out independently by 3 authors (AW, HS, DK). The primary outcome measure employed in this review was the number of 10-gram units of alcohol; wherever possible, reported outcomes were converted into this metric. Effect sizes were estimated using the pooled baseline standard deviation [33]. Differences between posttreatment or follow-up means of each group and their baseline mean were obtained. The change score for the control group was subtracted from the change score of the experimental group, and the result was divided by the pooled standard deviation. Where a full set of data was not provided, the calculation of the pre-post effect sizes between conditions employed the mean changes from baseline to posttreatment and their associated standard deviations. Where data was insufficient or not available in the published paper or by contacting authors, studies were excluded from the relevant analysis. 

## Results

### Description of Studies

**Figure 1:**
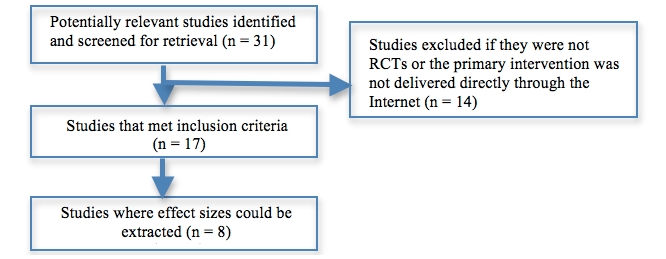
Study identification and analysis flow diagram

The literature search identified 31 studies, 17 of which were of online Internet alcohol intervention programs that met inclusion criteria (see Figure 1). After seeking clarification from the lead authors of the respective papers, 2 studies [8,34] were excluded from the analyses of effect sizes since the computer intervention was delivered on an intranet platform rather than via the Internet. A third study by the same research group was included [35] because the intervention was developed to be Web-based and was delivered online albeit accessed in the course of this specific study on student health clinic computers. Studies by Newton et al [36] and Turrisi et al [37] were also excluded from the primary outcome analyses because they included significant face-to-face components. Differential effect sizes were calculated for 8 of the 17 identified randomized controlled trials (RCTs).

#### Participants

Most studies that met inclusion criteria targeted university students (12/17 or 70.6%), although some recruited general company employees [38,39] or community members [40-42]. While the university-based studies generally involved participants aged 18 to 25, in other studies, the median reported age was 43.1 years. The size of recruitment pools and participation rates varied substantially. This appeared to be mediated in part by the study’s target population, the presence of incentives, the marketing and recruitment strategy, and whether participation was mandated (Table 1). In fact, Matano et al [38] distributed 8567 invitations to achieve 316 preintervention surveys.

Study sample sizes ranged from 40 to 3216 (median 196) with 64.7% (11/17) of the RCTs targeting at-risk, heavy, or binge drinkers. The percentage of females ranged from 27.6% to 77.9% (mean 54.5%, median 52%), which is substantially greater than in most AOD clinics (Table 1).

#### Interventions

Of the studies that met criteria, 70.6% (12/17) evaluated the impact of brief personalized feedback, and 41.2% (7/17) examined an online multi-module information/education treatment (often incorporating personalized feedback). Control groups typically received psychoeducational resources (10/17 or 58.8%) or completed an online assessment.

#### Duration of Trials

Posttreatment assessments were conducted anywhere from 1 week to 12 months posttreatment, with several studies conducting assessments at multiple time points. Across the 17 studies, 7 (47.1%) had a maximum follow up period of a month, 4 (23.5%) had a maximum 3-month follow up, and 3 (17.6%) followed participants to 6-months post intervention. Only Kypri et al [35] employed a 12-month follow up.

Reported retention rates in the intervention groups ranged from 38.9% to 100%, and in controls, from 33.4% to 100%. Median reported retention in the treatment condition was 83.4% at 1 month, 74.5% 3 months, and 74.5% at 6 months. In control groups, the median retention rates at the same time points were 80%, 70.4%, and 74.9%. The Kypri et al study [35] reported 12-month retention rates of 83.5% for the intervention group and 86.3% for the control condition.

Several studies reported only combined retention data. The studies by Doumas and Hannah [39] and Doumas et al [43] reported 1-month whole sample retention rates of 63.3% and 88.2% respectively, with the Walters et al [23] study reporting retention rates of 71.7% at 2 months and 77.4% at 4 months. The Motano et al [38] study reported whole sample retention rates of 83.8% at 3 months.

#### Outcomes

A wide variety of outcome assessments were employed across the studies with all studies including some measure of alcohol consumption (eg, unit grams of alcohol, number of standard drinks, or blood alcohol concentrations) in relation to either a typical drinking occasion or when the greatest amount was consumed on a single occasion. In many cases, the measure of frequency of alcohol consumption used was either 4 or more or 6 or more drinks per occasion or drinking to intoxication. Quantity and frequency measures related to a designated assessment period (a typical week, the previous week, 2 or 6 weeks, or up to the last 12 months). Several studies assessed alcohol use in relation to specific events (eg, 21^st^ birthdays [44], homecomings, holidays, or pub nights [45]). A number of studies included measures of personal, social, sexual, or legal consequences of drinking [34,36,46,47], protective factors [45], alcohol-related knowledge [19,36], readiness to change [40,45,48], intention to seek help [48], drinking related self–efficacy [46], or outcome expectancies [36,46].

**Table 1:** Characteristics of online alcohol-related randomized controlled trials

Author	Recruitment Pool	Description and Size of Intervention Group	Description and Size of Control Group	Age Reported Mean (SD) and/or Range (Years)	Percent Female Gender
Bewick et al [49]^a^	University students recruited through a student experience survey	Personalized normative feedback n = 234	Assessment only n = 272	Mean 21.3 (SD 3.7)	69
Chiauzzi et al [45]^b^	2^nd^ and 4^th^ year university students from 5 colleges who responded to local advertisement and subsequently screened as binge drinkers	MyStudentBody, a website that provides motivational feedback and alcohol-related resources n = 131	Alcohol and You, a website that provides educational material only n = 134	Mean 19.9 (SD 1.6)	54
Croom et al [19]	All incoming 1^st^ year university students	Participant survey, knowledge test, and online course n = 1608	Survey and knowledge test n = 1608	18 to 24	49.1
Cunningham et al [41]^b^	Problem drinkers identified through a general population telephone survey	Web-based personalized feedback (approximately 10 minutes) n = 92	List of alcohol education resources n = 93	Mean 40.1 (SD 13.4)	47
Doumas and Hannah [39]^c^	Workplace employees of 5 local companies	(1) Web-based feedback (approximately 15 minutes) n = 60 (2) Web-based feedback and motivational interviewing n = 63	Assessment only n = 73	18 to 24	73
Doumas et al [43]	University students mandated for alcohol counselling	Web-based personalized normative feedback (15 minutes) n = 46	Web-based education (approximately 45 minutes) n = 31	Mean 19.2 (SD 1.33) 18 to 24	27.6
Hester et al [40]	Newspaper advertisement recruiting heavy drinkers	Online alcohol education resource and Web-based alcohol moderation program n = 40	Access to online alcohol education resources n = 44	Intervention group mean 48.7; control group mean 52.1	56
Hustad et al [47]^b,d^	1^st^ year university students	(1) AlcoholEdu, 3-hour modularized program n = 26 (2) Alcohol eCHECKUP TO GO (eCHUG), 20-minute personalized normative feedback program n = 31	Assessment only n = 25	Mean 18.1 (SD 0.3)	51
Kypri et al [50]^b^	Heavy drinking university students majoring in psychology and attending university health care	Web-based motivational assessment and personalized feedback (10 to 15 minutes) n = 1251	Screening only n = 1184	Mean 19.7 (SD 1.8), 17 to 24	45.3

Kypri et al [35]^b^	Undergraduate university students, who scored ≥ 8 on Alcohol Use Disorders Identification Test (AUDIT)	(1) Multidose motivational intervention n = 145 (2) Single dose motivational intervention n = 138	Information pamphlet n = 146	Mean 20.1 (SD 2.2), 17 to 29	52
Matano et al [38]	Workplace employee website	Full individualized feedback regarding alcohol risk, information regarding alcohol use, and feedback regarding stress and coping n not specified	General information regarding alcohol and limited individualized feedback regarding stress and coping n not specified	Mean 39.9 (SD 11.3)	77.9
Moore et al [51]	Convenience sample of 1^st^ year university students enrolled in 3 college courses	Web-based binge-drinking intervention n = 59	Correspondence-based binge-drinking intervention n = 57	Mean 21.7 (SD 0.2), 18 to 25	57.8
Neighbors et al [44]^b^	University students turning 21 during 2 academic quarters who intended drinking 2 or more drinks on their birthday	Web-based personalized feedback n = 150	Assessment only n = 145	20 year olds	51.1
Riper et al [42]^b^	Advertisements in national newspapers and health-related websites recruiting adult problem drinkers	Web-based multi-component Cognitive Behaviour Therapy self-help intervention n = 130	Online psycho-educational alcohol use brochure n = 131	18 to 65, intervention group mean 45.9 (SD 8.9), control group mean 46.2 (SD 9.2)	49
Saitz et al [48]	1^st^ year university students identified as engaging in hazardous alcohol use ( ≥ 8 on AUDIT)	Extensive individualized brief feedback intervention n = 324	Individualised minimal brief intervention n = 326	18 and over	63.7
Walters et al [23]	1st year university students assessed within the study as “at risk” drinkers	eCHUG, personalized normative feedback program (20 minutes) n not specified	Assessment only n not specified	Not specified	48.1
Weitzel et al [46]	University students who self-identified as drinking more than 1 once of alcohol per week recruited through emails and on-campus advertising	Online daily diary and individualized tailored messages n = 20	Online daily survey n = 20	Mean 19.2, 18 and over	55

^a^ Shown are baseline sample size and data. Data shown for this study in Tables 2 include only participants available at posttreatment.

^b^ Intention-to-treat analysis was conducted on some or all measures.

^c^ This study included a second intervention condition which consisted of Web-based feedback as well as motivational interviewing (MI). However, the motivational interviewing component was delivered face-to-face rather than via the Internet and, therefore, the effect size data from the second intervention condition is not included in calculations of mean effect sizes.

^d^ Completion of AlcoholEdu program was a university-wide administrative requirement.

### Effects of Interventions

#### Alcohol Units

Based on 5 RCTs [41-43,47,49] where a measure of alcohol units per week or month could be extracted, differential effect sizes to posttreatment ranged from 0.02 to 0.81 (median 0.54) (Table 2). Using the full samples of participants, the mean differential effect size was 0.42. If only identified problem drinkers in the Cunningham study [41] are included (rather than the full sample dataset), the effect size rose to 0.47. The pre-post differential effect size for brief personalized feedback programs [41,43,47,49] ranged from 0.02 to 0.81 (mean 0.39, median 0.33), and for the multi-session modularized programs of Riper et al [42] and Hustad et al (AlcoholEdu) [47], a pre-post differential effect size of 0.56 was obtained in each case.

Employing Cohen’s effect size evaluation benchmarks [52], the effects on alcohol units consumed were generally in the small to medium range. A notable exception was the study by Hustad and colleagues [47] undertaken with a college sample. That study examined the effectiveness of 2 of the most commonly used electronic interventions for heavy drinking in college students: AlcoholEdu, an education style multi-session modularized Internet program, and Alcohol eCHECKUP TO GO (eCHUG), an intervention based on personalized normative feedback. In relation to peak drinks per occasion per month, the eCHUG personalized normative feedback intervention resulted in a large differential effect size (0.81).

Only 1 RCT allowed extraction of a follow-up effect size on alcohol units. The examination by Cunningham and colleagues [41] of the online CheckYourDrink.net website (10-minute online normative and alcohol severity feedback) resulted in a small differential effect size of 0.23 for the full sample at the 6-month follow up. When only the data from identified problem drinkers was included, a moderate differential effect size (0.43) was obtained.

#### Blood Alcohol Concentrations

Pre-post data on peak blood alcohol concentrations (BAC) were available from 2 RCTs [44,47] (Table 3) and resulted in a mean differential effect size of 0.66. The personalized normative feedback program of Hustad et al [47] for eCHUG and that of Neighbors et al [44] achieved differential effect sizes of 0.87 and 0.22 respectively. Interestingly, the eCHUG intervention produced a BAC effect size (0.88), comparable to that of the more extensive modularized 3-hour online alcohol program, AlcoholEdu tested in the same trial.

#### Other Outcome Measures

Differential effect sizes were extracted from 5 RCTs [39,43,44,49,51] in relation to number of drinks on 21^st^ birthday [44], units of alcohol per occasion [49], peak consumption [39,43], frequency of drinking to intoxication [39,43], or 30-day quantity of alcohol use [51] (Table 3). Most studies obtained small effect sizes on these measures. However, of note is the Moore et al [51] study that employed a control group that differed from the intervention group primarily in mode of delivery (via postal services) rather than in key content. In this study, there was a greater fall in 30-day quantity of alcohol use (number of drinks per occasion) for postal delivery than for Internet delivery (differential effect size = -0.26). This, however, reflected higher consumption by the postal control group at baseline (mean 3.15 vs mean 2.49): Posttreatment alcohol quantities were comparable across the groups (postal 2.51, Internet 2.53).

**Table 2:** Randomized controlled trials of online alcohol interventions

Study	Treatment Group (or Treatment Group 1 if More Than One Group)					Control Group					
	Correction for Alcohol Units^a^	n	Mean (SD) Pre	Mean (SD) Post	Mean at Follow Up	n	Mean (SD) Pre	Mean (SD) Post	Mean at Follow Up	Pre-Post Effect Size (*d)*	Pre-Follow Up Effect Size (*d*)
Bewick et al [49]^b^	0.80	138	^b^	9.62 (10.86)		179	^b^	11.88 (14.9)		0.02^b^	
Riper et al [42]^c^	1.00	130	43.7 (21.0)	28.7^d^		131	43.5 (22.3)	40.6^d^		0.56	
Doumas et al [43]^c^	1.40	46	11.42 (9.2)	6.8 (5.43)		31	9.86 (7.42)	8.1 (8.27)		0.33	
Hustad et al [47] (1) AlcoholEdu^c,e^	1.40	26	8.9 (11.62)	11.0 (15.54)		24	9.28 (12.4)	18.14 (17.25)		0.56	
Hustad et al [47] (2) eCHUG^c,e^	1.40	30	12.4 (14.29)	10.4 (11.09)		24	9.28 (12.4)	18.1 (17.25)		0.81	
Cunningham et al [41], full sample^c^	1.36	92	18.9 (14.82)	14.96 (12.38)	15.1 (12.1)	93	16.18 (13.7)	15.5 (14.0)	15.64 (14.0)	0.23	0.23
Cunningham et al [41], problem drinkers only	1.36	35	30.6 (17.14)	20.54 (15.23)	21.76 (16.2)	37	25.98 (16.3)	25.02 (16.73)	24.34 (17.0)	0.54	0.43

^a^ The table displays means in 10-gram alcohol units. Calculations use stated drink sizes where available. Where a paper referred only to numbers of drinks, these were adjusted using national “standard drink” sizes [53]. Alcohol units calculated per week unless otherwise stated

^b^ Baseline data presented by Bewick et al [49] is not from the posttreatment sample. The pre-post difference is based on the mean differences and related SDs from baseline to posttreatment. Analyses in that study were based on transformed data.

^c^ Means were calculated using identified comparisons.

^d^ Post SDs were not reported.

^e^ Units of alcohol reported are per month.

**Table 3:** Randomized controlled trials of online alcohol interventions: Effect sizes (d) obtained across blood alcohol concentrations and other alcohol-related measures

Study and Outcome Measure	Treatment Group (or Treatment Group 1 if More Than One Group)			Control Group			
	n	Mean (SD) Pre	Mean (SD) Post	n	Mean (SD) Pre	Mean (SD) Post	Pre-Post *d*
Hustad et al [47] (1) AlcoholEdu, peak BAC	26	0.08 (0.10)	0.08 (0.09)	24	0.07 (0.08)	0.15 (0.15)	0.88
Hustad et al [47] (2) eCHUG, peak BAC	30	0.08 (0.10)	0.08 (0.08)	24	0.07 (0.08)	0.15 (0.15)	0.87
Neighbors et al [44], peak BAC on 21st birthday	150	0.11 (0.10)	0.10 (0.11)	145	0.12 (0.11)	0.13 (0.13)	0.22
Neighbors et al [44], number of drinks on 21st birthday	150	7.23 (5.29)	6.4 (6.13)	145	7.14 (5.12)	7.00 (5.57)	0.13
Bewick et al [49], units per occasion	138	^a^	6.77 (4.54)	179	^a^	7.84 (5.78)	0.23^a^
Doumas and Hannah [39], frequency of drinking to intoxication (ie, number of times drunk or high from alcohol) during the past 30 days	60	1.44(2.06)	0.85 (1.63)	73	1.19 (1.70)	1.02 (1.88)	0.22
Doumas and Hannah [39], peak alcohol consumption (number of drinks consumed on the occasion on which the individual drank the most in the previous month)	60	5.12 (5.36)	3.55 (3.91)	73	4.15 (4.80)	3.98 (4.70)	0.28
Doumas et al [43], peak alcohol consumption (number of drinks consumed on the occasion on which the individual drank the most in the previous month)	46	8.77 (4.53)	6.95 (3.92)	31	6.21 (2.77)	5.88 (3.07)	0.38
Doumas et al [43], drinking to intoxication (ie, number of times drunk or high from alcohol during the past 30 days)	46	0.84 (0.37)	0.68 (0.47)	31	0.79 (0.41)	0.71 (0.46)	0.21
Moore et al [51]^b^, 30-day frequency of alcohol use	53	4.74 (5.82)	3.68 (4.95)	47	5.38 (5.83)	5.02 (4.94)	0.12
Moore et al [51]^b^, 30-day quantity of alcohol use (number of drinks per occasion)	53	2.49 (2.55)	2.53 (2.33)	47	3.15 (2.6)	2.51 (2.33)	-0.26

^a^ Baseline data presented by Bewick et al [49] is not from the posttreatment sample. The pre-post difference is based on the mean differences and related SDs from baseline to posttreatment. Data presented here are not transformed.

^b^ This study’s control group differed primarily in mode of delivery (via postal services) rather than in key content.

## Discussion

### Review of Findings

Internet interventions offer an alternative, accessible treatment option for people with alcohol-related problems. Their effectiveness, however, has not been systematically evaluated. To date, there have been a limited number of published RCTs of online alcohol interventions. The majority have been conducted with university or student populations and have employed a range of incentives and inducements to achieve an acceptable participation and retention rate. These groups tend to be young (early 20s) with a predominance of females. Given the high rates of binge drinking in this age group [7] and the fact that young people—particularly females [54]—are unlikely to access traditional face-to-face services, engagement of these students is an important achievement. However, caution should be exercised in generalizing from these findings, as student samples may not be representative of the general community on motivation, reading level, computer and Internet access, and computer literacy, among other factors.

The brevity of intervention descriptions in the published papers, variable intervention uptake and completion rates, and the heterogeneity of outcome measures and follow-up periods across studies impede the ability to generalize about the efficacy and utility of Internet-based interventions for alcohol use. Overall, online alcohol interventions (whether only involving brief personalized feedback or comprising multiple modules) appear to bring about small but meaningful differential reductions in 10-gram alcohol units consumed, blood alcohol concentrations, and a range of other alcohol-related measures. In particular, they appear more efficacious than assessment alone or general education about alcohol.

Of studies published to date, 3 stand out. Hustad and colleagues [47] undertook the only study to produce a large differential effect size (greater than or equal to 0.80) for the treatment group relative to the control. That study examined the effectiveness of 2 of the most commonly used electronic interventions for heavy drinking in college students: AlcoholEdu, an education style Internet program, and Alcohol eCHECKUP TO GO (eCHUG), an intervention based on personalized normative feedback. However, caution should be exercised in interpreting these results as 35% of the invited participants did not consent or respond to the invitation to participate in the study.

Moore and colleagues [51] compared the efficacy and feasibility of a binge drinking prevention program for college students delivered via the Internet or via postal mail. Both modes of delivery were efficacious in reducing drinking of students who were binge drinking at baseline, but there was no significant difference in outcome between the 2 delivery modes except for 30-day quantity of alcohol use, where the postal intervention was associated with a greater reduction but a similar posttreatment mean. Replication in samples with more comparable baseline scores is needed.

The trial of Riper and colleagues [42] is also worth highlighting. That study tested a multi-component, self-help intervention for problem drinkers. A moderate pre-post differential effect size on 10-gram alcohol units consumed per week was achieved. At posttreatment, 17.2% of the intervention group had reduced their drinking levels to within Dutch guidelines for low-risk drinking compared with 5.5% of control participants. Decreases in mean weekly alcohol intake (15 units per week) were substantially greater than those in the control group (2.9 units per week). However, only 45.4% of intervention participants made use of the online intervention, and only 51.1% of controls used the psychoeducational brochure.

### Conclusions

#### Implications for Research.

The use of online interventions for the treatment of alcohol-related problems requires more extensive research to establish the clinical appropriateness and usability of online health technologies [29], especially in nonstudent contexts. Given the potential benefit of these interventions for cost-effective delivery of interventions to large numbers of people, future research should incorporate economic analyses. As suggested by Copeland and Martin [24], the rigorous evaluation of online interventions would encourage their wider implementation and dissemination and increase their impact on public health and related service costs.

A significant challenge for this field is that advances in equipment, connectivity, and software capabilities are occurring much more rapidly than the evidence base can be fully established. In this context, recommendations for practice must necessarily rely to some extent on analogies from evidence that has been obtained on similar interventions using older forms of delivery. However, transfer of interventions to new modes of delivery run the risk of losing the key effective ingredients. It remains important that researchers respond rapidly to new technological advances, adapting treatments and routinely conducting trials to ensure that effects on alcohol use are retained.

As with all remotely delivered interventions, engagement of participants remains an issue. Internet-based interventions are likely to have greater reach if they are interfaced with targeted marketing campaigns or are embedded in routine primary care. Further research on the most effective marketing and widespread dissemination of these interventions is required.

#### Implications for Practice

While the current research evidence is fragmented and requires greater methodological rigor, it suggests that problematic or at-risk users may benefit from online alcohol interventions and that they may be a useful preventative and first step for groups such as women or young people who may not otherwise access more traditional AOD health services. Our confidence in these interventions is boosted by decades of research on bibliotherapies [55] and face-to-face interventions for alcohol use, including robust evidence in favour of brief interventions [56]. While further randomized controlled trials are required, there is sufficient evidence to suggest that standard health services and community campaigns evaluate and deploy online alcohol interventions to address alcohol-related problems.

## Abbreviations

AOD: alcohol and other drug
AUDIT: Alcohol Use Disorders Identification Test
BAC: blood alcohol concentrations
MI: motivational interviewing
RCT: randomized controlled trials
WHO: World Health Organization
